# Using disproportionality analysis to explore the association between periostitis and triazole antifungals in the FDA Adverse Event Reporting System Database

**DOI:** 10.1038/s41598-023-27687-0

**Published:** 2023-03-18

**Authors:** Hailong Li, Miao Zhang, Xuefeng Jiao, Yu Zhu, Yan Liu, Linan Zeng, Huiqing Wang, Lei Zhang, Wei Zhang, Lingli Zhang

**Affiliations:** 1 Department of Pharmacy, West China Second University Hospital, Sichuan University, Chengdu, China; 2 Evidence-Based Pharmacy Center, West China Second University Hospital, Sichuan University, Chengdu, China; 3 NMPA Key Laboratory for Technical Research On Drug Products In Vitro and In Vivo Correlation, Chengdu, China; 4 Key Laboratory of Birth Defects and Related Diseases of Women and Children, Ministry of Education, Sichuan University, Chengdu, China; 5 West China School of Pharmacy, Sichuan University, Chengdu, China; 6 Department of Pediatrics, West China Second University Hospital, Sichuan University, Chengdu, China; 7 College of Computer Science, Sichuan University, Chengdu, China; 8 West China Biomedical Big Data Center, West China Hospital, Sichuan University, Chengdu, China; 9 Medical Big Data Center, Sichuan University, Chengdu, China

**Keywords:** Fungal infection, Drug safety

## Abstract

Though triazole antifungals are the first choice for preventing and treating invasive fungal infections, periostitis caused by voriconazole has been described in emerging case reports; however, no studies exist on this association in real-world clinical settings. Our study aimed to identify the association between periostitis and triazole antifungals by analyzing data from the FDA Adverse Event Reporting System (FAERS). We extracted and analyzed reports on the association between periostitis and triazole antifungals in FAERS from the first quarter of 2004 to the second quarter of 2022 using OpenVigil 2.1. Disproportionality analysis was performed to evaluate the association between periostitis and triazole antifungals, and chi-squared (χ^2^), relative reporting ratio (RRR), reporting odds ratio (ROR), proportional reporting ratio (PRR), and Bayesian confidence propagation neural networks (BCPNN) of information components (IC) were reported. In total, 143 patients experienced periostitis while using voriconazole. Disproportionality analysis identified an association between periostitis and voriconazole (χ^2^ = 82,689.0, RRR = 583.6, 95%CI [472.4, 721.1], PRR = 1808.9, 95%CI [1356.0, 2412.9], ROR = 1831.7, 95%CI [1371.6, 2446.3], IC = 9.2, 95%CI [8.6, 9.8]). However, no safety signals were observed between periostitis and other triazole antifungals. When stratified by sex and age, disproportionality analysis identified positive signals between periostitis and voriconazole. The possible association between periostitis and voriconazole should attract sufficient attention in clinical practice. Alternative treatment with other triazole antifungals can be considered, and causality needs to be verified in further prospective studies.

## Introduction

Triazole antifungals are the first choice for preventing and treating invasive fungal infections. In addition, they are recommended for prophylactic treatment or general antifungal prophylaxis in patients undergoing solid organ and hematopoietic stem cell transplantation^1^. Generally, triazole antifungals must be taken for long periods (weeks to months), followed by long-term suppressive therapy^2^. However, the long-term use of triazole antifungals has raised concerns about cost, development of resistance, and treatment-related adverse effects. Meanwhile, with the increasing use frequency of triazole antifungal drugs, their safety is receiving more and more attention. Common adverse events with voriconazole include visual impairment, fever, rash, nausea, vomiting, diarrhea, headache, septicemia, peripheral edema, abdominal pain, and respiratory dysfunction. Periostitis caused by voriconazole therapy has been described in various case reports^3–12^. Symptoms of periostitis typically include myalgia or diffuse pain in one or more skeletal areas, often involving the tibia, orbital bone, or root bone. A case report published in *The New England Journal of Medicine* revealed that a patient with voriconazole-induced periostitis had reduced pain at a 7-month follow-up after discontinuation of voriconazole, but the range of motion limitations and bone swelling persisted in the hand^13^. In cases of infectious periostitis, sepsis may occur and involve all body organs^14^. In addition, it has been suggested that periostitis is mainly caused by the fluoride in voriconazole, and alternative therapy with other triazole antifungals can be clinically considered to avoid periostitis^15^. However, there have been no studies on the association between periostitis and triazole antifungals in a large population, including signal mining studies based on the adverse event reporting database. Therefore, it is unclear whether there is an association between periostitis and triazole antifungals in real-world clinical settings.

The FDA Adverse Event Reporting System (FAERS) is a database utilized for collecting information on spontaneously reported adverse drug events. Due to the large amount of data, diversity of data information, and free access to the public, it is often used in studies of adverse drug event signal mining^16^. FAERS receives approximately 1.5 million reports of adverse events about drugs and medical devices every year and can reflect adverse events in real-world clinical settings. In addition, data mining algorithms have already been developed for signal detection in this database; for example, a “positive signal” refers to a statistical association between an adverse event and a drug^17^. Thus, FAERS is now widely used to detect potential drug-associated adverse events^18,19^.

Therefore, in this study, we identified the possible association between periostitis and triazole antifungals based on the FAERS database. This study can provide research hypotheses on the causality between triazole antifungal drugs and periostitis for further studies, and can also provide evidence for optimizing treatment, prevention, and response to adverse drug events.

## Results

### Characteristics of patients taking triazole antifungals and experiencing periostitis

We found 143 patients taking voriconazole and experiencing periostitis**.** No relevant reports on fluconazole, itraconazole, posaconazole, and ravuconazole were available. Detailed characteristics of the patients are shown in Table 1. For voriconazole, 46.85% of the patients with periostitis were male, 41.26% were female, and 11.89% were unknown. There were 4 patients (2.80%) aged less than 18 years, 87 patients (60.84%) aged 18–65 years, and 31 patients (21.68%) aged more than 65 years. The largest number of patients was reported between 2019 and 2022, with 62 patients (43.36%). The United States had the highest number of reports among all reporting countries, with 89 patients (62.24%). Among all outcomes, other outcomes (46.15%) accounted for the largest proportion, followed by hospitalization (24.48%), unknown (20.28%), disability (5.59%), death (2.10%), and life-threatening (1.40%). Infection accounted for the largest proportion (36.36%) of all the indications. In addition, for fluconazole and itraconazole, the three patients that emerged were included in the 143 patients taking voriconazole at the same time. Therefore, except for voriconazole, triazole antifungals have no data on the occurrence of periostitis.

**Table 1:** Characteristics of patients with voriconazole-induced periostitis.

Characteristics		Patients with voriconazole-induced periostitis
Overall		143
Sex	Male (n, %)	67 (46.9%)
	Female (n, %)	59 (41.3%)
	Unknown	17 (11.9%)
Age	< 18 years	4 (2.8%)
	18–65 years	87 (60.8%)
	> 65 years	31 (21.7%)
	Unknown	21 (14.7%)
Year	2007–2010	6 (4.2%)
	2011–2014	28 (19.6%)
	2015–2018	47 (32.9%)
	2019–2022	62 (43.4%)
Report countries	United States	89 (62.2%)
	Canada	13 (9.1%)
	Australia	8 (5.6%)
	France	6 (4.2%)
	Denmark	5 (3.5%)
	Germany	5 (3.5%)
	Netherlands	3 (2.1%)
	United Kingdom	2 (1.4%)
	India	2 (1.4%)
	Argentina	1 (0.7%)
	Belgium	1 (0.7%)
	Spain	1 (0.7%)
	New Zealand	1 (0.7%)
	Unknown	6 (4.2%)
Outcome	Death	3 (2.1%)
	Hospitalization	35 (24.5%)
	Life-threatening	2 (1.4%)
	Disability	8 (5.6%)
	Other	66 (46.2%)
	Unknown	29 (20.3%)
Indication	Infection	52 (36.4%)
	Aspergillosis	39 (27.3%)
	Prophylaxis	17 (11.9%)
	Inflammation	6 (4.2%)
	Antifungal treatment	2 (1.4%)
	Mycetoma mycotic	1 (0.7%)
	Immunosuppression	1 (0.7%)
	Cystic fibrosis	1 (0.7%)
	Unknown	24 (16.8%)

### The association between periostitis and triazole antifungals

For the 143 patients with voriconazole-related periostitis, χ^2^ was 82,689.0, relative reporting ratio (RRR) was 583.6, 95% confidence interval (CI) [472.4, 721.1]; proportional reporting ratio (PRR) was 1808.9, 95% CI [1356.0, 2412.9]; reporting odds ratio (ROR) was 1831.7, 95% CI [1371.6, 2446.3]; and Bayesian confidence propagation neural networks (BCPNN) of information components (IC) was 9.2, 95%CI [8.6, 9.8] (Table 2), which met the criteria for positive signals in disproportionality analysis. Therefore, there may be an association between periostitis and voriconazole. No association was observed between periostitis and other triazole antifungals.

**Table 2:** The association between periostitis and voriconazole.

	Voriconazole
N	143
χ^2^	82,689.0
RRR (95%CI)	583.6 (472.4, 721.1)
PRR (95%CI)	1808.9 (1356.0, 2412.9)
ROR (95%CI)	1831.7 (1371.6, 2446.3)
IC (95%CI)	9.2 (8.6, 9.8)

*RRR* relative reporting ratio, *CI* confidence interval, *PRR* proportional reporting ratio, *ROR* reporting odds ratio, *IC* information components.

### The association between periostitis and voriconazole in different sexes

When stratified by sex, the number of voriconazole-related periostitis reports in males was 67, χ^2^ was 29,713.6, RRR was 451.4, 95% CI [327.8, 621.6]; PRR was 2226.4, 95% CI [1308.3, 3788.9]; and ROR was 2251.9, 95% CI [1321.5, 3836.5], while the number of voriconazole-related periostitis reports in females was 59, χ^2^ was 45,751.2, RRR was 790.2, 95% CI [574.7, 1086.5]; PRR was 1848.4, 95% CI [1252.6, 2727.6]; and ROR was 1877.7, 95% CI [1269.1, 2778.1] (Table 3). Thus, there may be an association between periostitis and voriconazole, regardless of sex.

**Table 3:** The association between periostitis and voriconazole by sex.

	N	χ^2^	RRR (95%CI)	PRR (95%CI)	ROR (95%CI)	IC (95%CI)
Male	67	29,713.6	451.4 (327.8, 621.6)	2226.4 (1308.3, 3788.9)	2251.7 (1321.5, 3836.5)	8.8 (8.4, 9.1)
Female	59	45,751.2	790.2 (574.7, 1086.5)	1848.4 (1252.6, 2727.6)	1877.7 (1269.1, 2778.1)	9.6 (9.2, 9.9)

*RRR* relative reporting ratio, *CI* confidence interval, *PRR* proportional reporting ratio, *ROR* reporting odds ratio, *IC* information components.

### The association between periostitis and voriconazole in different age groups

When stratified by age, the number of voriconazole-related periostitis reports in patients less than 18 years was 4, χ^2^ was 582.0, RRR was 191.7, 95% CI [56.2, 653.8]; PRR was 446.1, 95% CI [100.0, 1990.0]; and ROR was 448.1, 95% CI [100.1, 2005.4]. The number of voriconazole-related periostitis reports in patients aged 18–65 years was 87, χ^2^ was 39,262.2, RRR was 457.8, 95% CI [348.7, 601.0]; PRR was 1503.7, 95% CI [1028.3, 2199.0]; and ROR was 1531.7, 95% CI [1045.1, 2244.6]. Finally, the number of voriconazole-related periostitis reports in patients more than 65 years was 31, χ^2^ was 14,057.9, RRR was 469.7, 95% CI [292.7, 753.7]; PRR was 2545.2, 95% CI [1121.7, 5775.4]; and ROR, 2573.1, 95% CI [1132.1, 5848.2] (Table 4). Thus, there may be an association between periostitis and voriconazole at different ages.

**Table 4:** The association between periostitis and voriconazole by age.

Age,years	N	χ^2^	RRR (95% CI)	PRR (95% CI)	ROR (95% CI)	IC (95% CI)
< 18	4	582.0	191.7 (56.2, 653.8)	446.1 (100.0, 1990.0)	448.1 (100.1, 2005.4)	7.5 (5.7, 8.6)
18–65	87	39,262.2	457.8 (348.7, 601.0)	1503.7 (1028.3, 2199.0)	1531.7 (1045.1, 2244.6)	8.8 (8.4, 9.1)
> 65	31	14,057.9	469.7 (292.7, 753.7)	2545.2 (1121.7, 5775.4)	2573.1 (1132.1, 5848.2)	8.9(8.3, 9.3)

*RRR* relative reporting ratio, *CI* confidence interval, *PRR* proportional reporting ratio, *ROR* reporting odds ratio, *IC* information components.

## Discussion

To our knowledge, this is the first study to investigate the association between periostitis and triazole antifungals by analyzing FAERS data. Our study observed 143 patients with adverse event reports of voriconazole-related periostitis, whereas no reports were found between periostitis and other triazole antifungals. The results of the subgroup analysis suggested that voriconazole may be associated with periostitis, regardless of sex and age. The reason for the large values of RRR, PRR, ROR may be that the proportion of voriconazole-associated periostitis cases were much higher than that of other drugs. This also indicated that voriconazole is probably associated with periostitis. The wide confidence intervals of PRR, RRR, and ROR is likely because of the extremely low proportion of reports with periostitis.

Recently, emerging case reports have raised awareness of periostitis, a potentially fatal complication associated with voriconazole^12,20^. No case reports have described periostitis caused by other triazole antifungals^15^. Our study validated the findings of these case reports by identifying an association between periostitis and voriconazole. Voriconazole, a trifluoride antifungal, is believed to cause periostitis by increasing circulating levels of fluoride following voriconazole metabolism or by direct effects of the fluoride component of voriconazole^11,14,21–23^. Voriconazole-related periostitis usually occurs after high-dose (median 600 mg/day) or prolonged (median 5.6 months) voriconazole therapy during transplantation. The main features of the diagnosis include diffuse bone pain, white spots on the teeth, elevated serum alkaline phosphatase and plasma fluoride levels, positive nuclear bone scans, and radiological findings. Under the circumstances, discontinuing voriconazole is the primary method of resolving bone pain. Case studies have shown that patients with periostitis who were switched from voriconazole to itraconazole experienced rapid relief of bone pain^6,11^. Previous studies have also suggested that periostitis may be related to the fluoride in voriconazole. Therefore, due to the absence of fluoride in itraconazole and the low fluoride content in posaconazole or itraconazole, voriconazole can be replaced by these triazole antifungals if clinically required^15^.

Our study has several strengths. First, this is the first study to detect the signals between periostitis and triazole antifungals using FAERS data, which could provide valuable evidence for further studies and clinical practice in this field. Second, the risks of periostitis in different sex and age groups are unclear, and subgroup analyses were conducted to identify sex-specific and age-specific adverse events associated with voriconazole. Third, FAERS includes all reports of adverse events submitted to the FDA, with a sample size large enough to identify rare adverse events that are difficult to detect in traditional epidemiological studies^25^.

However, several limitations of the present study must be considered when interpreting the results. First, the FAERS database is a spontaneous reporting adverse event database, which is limited by the initiative, accuracy, and timeliness of reporting adverse reactions by physicians, patients, and other healthcare providers; therefore, there may be the possibility of under-reporting and misreporting. Second, there are problems with non-standard reports and missing data in FAERS, such as age and sex. Third, most reporters come from the United States, the United Kingdom, and other countries. Thus, there are limitations in the generalization of conclusions among Asians. Fourth, the causality between periostitis and voriconazole is difficult to determine and needs to be verified in future prospective studies.

There may be an association between periostitis and voriconazole, which should attract sufficient attention in clinical practice. Alternative treatment with other triazole antifungals, such as posaconazole and itraconazole, can be considered clinically, and the causality between periostitis and voriconazole needs to be verified in future prospective studies.

## Methods

### Data source

FAERS is a spontaneous reporting adverse event database submitted by physicians, pharmacists, manufacturers, patients, and other healthcare providers that provides information on adverse events and medicine error reports^24^. The FAERS data included patient demographics, administrative information, drug treatment information, adverse events, and patient outcomes^25^. OpenVigil 2.1 is a pharmacovigilance data extraction, cleaning, mining, and analysis tool for the FAERS database^26^. Relevant information was extracted from FAERS from the first quarter of 2004 to the second quarter of 2022 using OpenVigil 2.1 software.

### Query construction

OpenVigil 2.1 mapped drug names (generic names, brand names, and abbreviations) to unique drug names using Drugbank and Drugs@FDA^27^. First, we identified the target drugs in the triazole antifungal family (fluconazole, itraconazole, voriconazole, posaconazole, and ravuconazole). We then extracted adverse event reports that included one or more triazole antifungals. The sex and age of patients' data were limited in the subgroup analysis.

### Definition of periostitis-related adverse events

In FAERS, adverse events are coded using preferred terms (PTs) in the Medical Dictionary for Regulatory Activities (MedDRA) terminology. We ultimately selected periostitis as an adverse event based on the structural hierarchy of the MedDRA terminology.

### Statistical analysis

Descriptive analysis was performed to characterize the features of patients taking triazole antifungals and experiencing periostitis as an adverse event. Disproportionality analyses were performed by calculating RRR, ROR, PRR, PRR, and BCPNN of IC with their 95% CIs to evaluate the association between adverse events and drugs^24,28^. Disproportionality analysis is commonly used by the World Health Organization for pharmacovigilance when analyzing post-marketing surveillance databases to evaluate the association between adverse events and drugs^29–31^. Moreover, disproportionality analysis is based on a four-fold table to detect potential adverse drug reaction signals by comparing the proportion of target events occurring for the target drug with the proportion of target events occurring for all other drugs (Table 5). According to the criteria outlined by Evans et al.^32^, a positive signal was defined as more than three patients with chi-squared (χ^2^) greater than four and PRR greater than two. First, we performed a disproportionality analysis to evaluate the association between periostitis and triazole antifungals. Next, considering that the risks of periostitis in different sex and age groups were unclear, we performed subgroup analyses by sex (male and female) and age groups (< 18 years old, 18–65 years old, and > 65 years old)^33–36^.

**Table 5:** 2 × 2 contingency table for disproportionality analysis.

	Drug(s) of interest	All other drugs	Total
Adverse event(s) of interest	a	b	a + b
All other adverse events	c	d	c + d
Total	a + c	b + d	a + b + c + d

“a” is the number of reports with adverse events of interest of the drugs of interest, “b” is the number of reports with adverse events of interest of all other drugs, “c” is the number of reports with all other adverse events of the drugs of interest, and “d” is the number of reports with all other adverse events of all other drugs.

(1) $$\upchi 2 = \frac{{(ad-bc)}^{2}(a+b+c+d)}{(a+b)(c+d)(a+c)(b+d)}$$ (2) $$\mathrm{RRR}=\frac{\mathrm{a}/(\mathrm{a}+\mathrm{b})}{\mathrm{c}/(\mathrm{c}+\mathrm{d})}$$ (3) $$\mathrm{PRR}=\frac{\mathrm{a}/(\mathrm{a}+\mathrm{c})}{\mathrm{b}/(\mathrm{b}+\mathrm{d})}$$ (4) $$\mathrm{ROR}=\frac{\mathrm{a}/\mathrm{c}}{\mathrm{b}/\mathrm{d}}$$ (5) $$\mathrm{IC}={log}_{2}\left(\mathrm{RRR}\right)$$.

### Ethics statement

The data sets are de-identified. There was no direct human participation in this study. Institutional Review Board requirements do not apply under 45 CFR 46.102. All experiments were performed in accordance with relevant guidelines and regulations.

## Data Availability

The datasets generated and/or analyzed during the current study are available from the corresponding author on reasonable request.
